# Do agronomic approaches aligned to regenerative agriculture improve the micronutrient concentrations of edible portions of crops? A scoping review of evidence

**DOI:** 10.3389/fnut.2023.1078667

**Published:** 2023-07-12

**Authors:** Muneta Grace Manzeke-Kangara, Edward J. M. Joy, R. Murray Lark, Sally Redfern, Ans Eilander, Martin R. Broadley

**Affiliations:** ^1^Division of Agricultural and Environmental Sciences, School of Biosciences, Sutton Bonington Campus, University of Nottingham, Loughborough, United Kingdom; ^2^Rothamsted Research, Department of Sustainable Soils and Crops, Harpenden, United Kingdom; ^3^Faculty of Epidemiology and Population Health, London School of Hygiene and Tropical Medicine, London, United Kingdom; ^4^Unilever Research and Development, Colworth Science Park, Bedford, United Kingdom; ^5^Unilever Research and Development, Unilever Foods Innovation Centre, WH Wageningen, Netherlands

**Keywords:** alliums, beta-carotene, iron, rice, tomato, vitamin C, wheat, zinc

## Abstract

Regenerative Agriculture (RA) is used to describe nature-based agronomic approaches that aim to build soil health and crop resilience, minimize negative environmental outcomes, and improve farmer livelihoods. A benefit that is increasingly attributed to crops grown under RA practices is improved nutritional content. However, we do not know the extent to which RA influences crop nutritional quality and under what management approaches and context, can such effects be realized. A scoping review of recent literature (Web of Science, 2000–2021) was carried out to assess the evidence that RA approaches improve crop micronutrient quality. Papers included combinations of agronomic approaches that could be defined as Regenerative: “Organic Inputs” including composts and manures, cover crops, crop rotations, crop residues and biochars; “Reduced Tillage”, “Intercropping”, “Biostimulants” e.g. arbuscular mycorrhizal fungi; plant growth promoting bacteria, and “Irrigation”, typically deficit-irrigation and alternate wetting and drying. The crop types reviewed were predetermined covering common sources of food and included: Tomato (*Solanum lycopersicum L.*), Wheat (*Triticum aestivum L.*), Rice (*Oryza sativa L.*), Maize (*Zea mays L.*), Pulses (Fabaceae), Alliums (*Allium spp.*), and “other” crop types (30 types). This scoping review supports a potential role for RA approaches in increasing the concentrations of micronutrients in the edible portions of several crop types under specific practices, although this was context specific. For example, rice grown under increased organic inputs showed significant increases in grain zinc (Zn) concentration in 15 out of 16 studies. The vitamin C concentration of tomato fruit increased in ~50% of studies when plants were grown under increased organic inputs, and in 76% of studies when plants were grown under deficit irrigation. Overall, the magnitude and reproducibility of the effects of RA practices on most crop nutritional profiles were difficult to assess due to the diversity of RA approaches, geographical conditions, and the limited number of studies for most crops in each of these categories. Future research with appropriate designs, improved on-farm surveillance and nutritional diagnostics are needed for better understanding the potential role of RA in improving the quality of food, human nutrition, and health.

## Introduction

Regenerative Agriculture (RA) is widely used to describe agronomic approaches based on the principles of improving soil health and sequestering carbon. Giller et al. (1) provide a comprehensive overview on RA from an agronomic perspective. Their review highlights that—ever since the RA term entered regular usage in the 1980s—RA is an evolving conceptual area which spans across agronomic, biophysical, and social justice dimensions. RA is typically framed in terms of agricultural systems which: (1) minimize the external impacts of agriculture beyond the farm; (2) minimize energy and other inputs into the farm; (3) sequester carbon, improve nutrient cycling and wider ecosystem services, (4) increase biodiversity, and (5) promote social justice. Soil conservation is considered as the entry point for most agronomic approaches informed by RA, although universally accepted formal definitions and inclusion criteria for RA are lacking (1).

**Figure 1:**
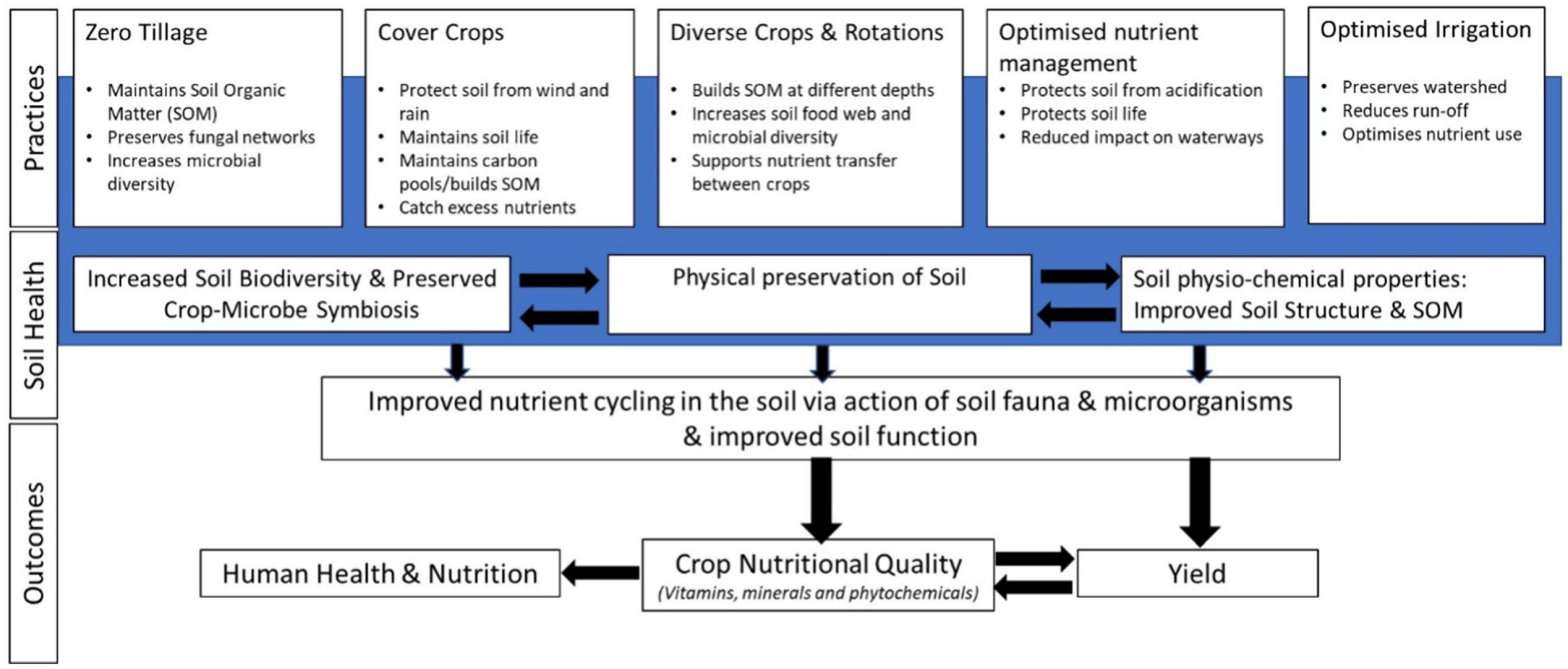
Interaction between Regenerative Agriculture practices, soil health, and nutritional quality.

Agronomic approaches within the scope of RA fall broadly into the following, non-exclusive, categories: (1) increased use of organic inputs^1^ for nutrition and soil cover, including animal manures, green manures/mulches/cover crops, crop rotations, other composts, and biochars; (2) reduced soil tillage, and (3) increased plant diversity (e.g., intercropping, more diverse rotations, agroforestry). The role of agrochemicals and genetically modified organisms (GMOs) within RA approaches remains contested. Some proponents of RA consider that the judicious use of these conventional and/or novel technologies can be consistent with the principles of RA. Other authors consider RA to align more closely with the principles of “organic” agriculture, although there are no current standards or certification for RA. Under conditions of zero tillage, for example, weed control using herbicides would be considered essential, whereas tillage would be used to control weeds in certified organic systems. Other agronomic strategies which are potentially consistent with RA principles include the use of biostimulants, such as incorporation of arbuscular mycorrhizal fungi (AMF), plant growth-promoting bacteria (PGPB), humic acids, and other beneficial bioactive compounds. The use of RA approaches such as organic inputs and reduced tillage to improve the functional attributes of soil microbiota indirectly, should also be considered (2). Similarly, water conservation techniques such as deficit irrigation systems can improve soil structure and reduce greenhouse gas losses in some cropping systems. This therefore reduces pressure on water availability in the landscape, and thereby be considered to fall within the scope of RA. Holistic RA systems which include grazing livestock can also be consistent with RA principles, including as a source of organic inputs. However, this area is contested due to the contributions of livestock to greenhouse gas emissions (1). From a nutritional yield perspective, the contribution of livestock to protein and micronutrient supply into food systems is still critical in many food system contexts. Recent evidence showed a high prevalence in selenium (Se) deficiency in cattle in Ethiopia, which negatively affects the health and productivity of livestock and consequently the soil-feed-livestock-human nexus (3). Detailed interactions between RA principles, soil health and nutritional quality are presented in Figure 1.

Giller et al. (1) define the two main challenges for RA as needing to: (1) restore soil health, including the capture of carbon to mitigate climate change, and (2) reverse biodiversity loss. There are implicit human health outcomes arising from climate change mitigation and improved biodiversity. Similarly, reductions in the intensive use of agrochemical inputs such as fertilizers and pesticides can also deliver potential health benefits on-farm during handling and application, and beyond the farm-gate. This can be directly, during processing and consumption; and through decreased potential for environmental pollution of landscapes (soil, water and atmosphere).

A further linkage between RA and human health can arise if these agronomic approaches lead to improvements in crop nutritional quality. Nutritional quality is the value of the product for the consumer’s physical health, growth, development, reproduction and psychological or emotional well-being (4). Here, we use the term crop nutritional quality to represent the nutritional value of the edible portions of crops. Crop nutritional quality is important because of the widespread global risks of micronutrient deficiencies (MNDs) which are likely to affect more than two billion people worldwide (5, 6). These MNDs, also known as “hidden hunger,” remain a major challenge for achieving the United Nations’ Sustainable Development Goal 2 (SDG2, zero hunger) by 2030 (7). Causes of MNDs include the inadequate dietary intakes of micronutrients, for example, calcium (Ca), iron (Fe), magnesium (Mg), iodine (I), Se, zinc (Zn) and vitamin A. Although MNDs can affect all people in all countries, the risks of MNDs are greater in low- and middle-income countries (LMICs) than in high-income countries [HICs; (5, 8, 9)]. In LMICs, access to foods from plant and animal sources that are richer in micronutrients is often limited and diets are dominated by cereals which typically have smaller micronutrient concentrations per unit energy (10). Although the bran and embryo fractions of cereal grains are often removed during milling, cereal grains also contain large concentrations of anti-nutritional compounds such as phytates (inositol phosphate compounds), which inhibit the absorption of Ca, Fe, Mg and Zn in the human gut (5). The consequences of MNDs include impaired physical and mental development and performance, and increased risks of communicable and non-communicable disease and mortality (7).

### Effects of different agronomic management practices on crop nutritional quality

The intercropping studies of Fusou Zhang and colleagues provide a detailed insight of the effects of an RA approach on crop nutritional quality, albeit generally from a crop nutrition, rather than a human nutrition perspective [reviewed by Zuo and Zhang (11)]. Intercropping is a production system in which two or more crop species are grown together in the same field, and contrasts with the more common monocropping systems where a single crop is grown. Intercropping systems such as peanut (*Arachis hypogaea* L.)/maize (*Zea mays* L.), wheat (*Triticum aestivum* L.)/chickpea (*Cicer arietinum* L.), and guava (*Psidium guajava* L.)/sorghum [*Sorghum bicolor* (L.) Moench] or maize, have been shown to improve Fe and Zn nutrition of crops leading to improved growth (11). The scientific principles behind this effect of intercropping are that “graminaceous” cereal crops can mobilize soil Fe and Zn through the release of siderophores and other compounds from their roots, which in turn will increase the availability of soil Fe and Zn to their neighboring “non-graminaceous” crop plant [reviewed for Fe, by Dai et al. (12)]. However, there is much still to learn about the role of specific compounds and their interactions with soil micro-organisms (microbiomes) in the rhizosphere and wider bulk soil, including how this might translate into agronomically appropriate combinations of crop types to yield nutritional benefits.

More recently, surveys of cereal grain quality among maize-based (13, 14) and wheat-based (15) smallholder farmers in sub-Saharan Africa (SSA) reported that use of animal and green manures led to nutritionally significant increases in grain Fe and Zn concentrations in maize. Similarly, augmented use of Integrated Soil Fertility Management (ISFM) approaches, including the use of organic inputs of leaf litter and cattle manure and micronutrient fertilizers improved grain Zn concentration in field-grown maize and cowpea (16, 17). These increases in grain Fe and Zn quality were likely greater than would have been expected from the direct effects of the additional inputs of Fe and Zn from the organic inputs into the system and are likely due to improvements in soil structure and retention/plant availability of micronutrients. The mechanisms by which soil organic carbon (SOC) affects crop Fe and Zn availability for uptake is not yet known, although it can help in terms of more effective micronutrient management of crops. For example, increased SOC supports better soil structure, including water and soil nitrogen (N) retention and provisioning to crops (18). Crops of better N status are likely to produce greater amounts of Fe- and Zn-chelating compounds such as nicotianamine synthase (NAS) and therefore support enhanced remobilization of Fe and Zn from crop leaves into grains, which is where Zn-protein co-localization occurs (19–23). Improved N nutrition of field-grown crops has been linked to increases in grain Zn concentrations in maize and cowpea [*Vigna unguiculata* (L.) Walp; (17)] and to increases in grain Fe concentration in field-grown finger millet [*Eleusine coracana* Gaertn; (24)]. However, synergies between crop N nutrition and grain Fe and Zn concentration are not seen consistently across all crop types under field conditions. This implies that complex interactions between yield improvements due to N and potential “dilution” effects on grain micronutrient concentration are likely to arise.

In a recent study from the US, a “paired-farm” approach was adopted to compare the nutritional quality of different crops [pea, *Pisum sativum* L.; sorghum; maize/corn; soybean, *Glycine max* (L.) Merr.] grown on RA farms with a proximal non-RA farm (25). There was evidence that crops grown on the RA farms exhibited greater concentrations of a range of vitamins and minerals in general than crops from non-RA farms, which corresponded with improvement in SOC on the RA farms. For example, maize, soybean, and sorghum grown on RA farms had 17, 22, and 23% more Zn than the same crops from a non-RA farm, albeit from a single contrast. The authors also noted potential decreases in the Zn concentration of cabbage (*Brassica oleracea* L. var. *capitata*) and pea, and, more importantly, that the paired-farm study design lacked statistical power to test for effects on individual crops. In the same paper, the use of cover crops vs. traditional fallow with regular herbicide use on no-tilled wheat resulted in significant increases in grain mineral micronutrient concentrations in wheat from the cover cropped field. A 48, 29, and 56% increase in grain Ca, Mg and Zn concentration was also reported, respectively. However, this was based on technical replication derived from a single sample (25).

### Soil health and crop nutritional quality

Relationships between soil health and food nutritional quality have recently been reviewed by Bourne et al. (26). There was no evidence of increased wheat grain protein concentrations under no-till conditions compared to conventional tillage from longer term studies. From shorter-term studies, there was considerable year-to-year variation and results from wheat studies were inconclusive. The authors noted that increases in SOC and soil organic N did not always result in increased grain N/protein concentration. Furthermore, there was some evidence of decreased grain protein concentration which could arise due to soil cultivation increasing soil N mineralization and availability to the crop. In crop rotational systems using legumes, increases in wheat grain protein concentration were observed, likely due to enhanced mineralization of N from residues from the preceding legume crop, and subsequent availability for uptake. For mineral micronutrients in wheat grain, the results were inconsistent across both tillage and rotational studies. However, several studies reported positive linkages between increased SOC, soil total N, and crop Zn uptake. This may be due to increased synthesis of Zn chelating compounds in crops and/or increases in soil cation exchange capacity and retention of Zn in plant available forms. However, Bourne et al. (27) noted these processes are complex. Furthermore, increased wheat grain Zn concentrations were associated with crop rotations that increased colonization of wheat roots by AMF and other fungi, for example by using clover (*Trifolium* spp.) or flax/linola (*Linum usitatissimum* L.) rather than canola (*Brassica napus* L.). Many studies on tomato (*Solanum lycopersicum* L.) quality lacked data on soil properties and yield, and focused more on secondary compounds (e.g., beta-carotene, lycopene, phenolics, vitamin C) rather than mineral micronutrients, in contrast to most wheat studies. Few consistent effects of production systems on mineral micronutrients and secondary compounds were noted, and these were likely influenced by genotypic differences between tomato varieties. Overall, few studies contained sufficient relevant information to provide evidence of linkages between metrics of soil health and crop nutritional quality of relevance to human health (26). Taken together, the evidence on linkages between agronomic practices which fall within the scope of RA and crop nutritional quality is currently limited. The authors of all these studies have noted the challenges in synthesizing data due to the many different input variables and geographies represented by these studies.

### Agricultural systems and crop nutritional quality

Dangour et al. (28) conducted a systematic review on the nutritional quality of crops grown in organic production systems. They found little evidence for differences in the nutritional quality of organic foods compared to conventionally produced foods. From an initial screen of 52,471 articles, and a shortlist of 162 studies (137 crops and 25 livestock products), only 55 articles were of satisfactory quality for a comparison of nutritional quality between organically- and conventionally-produced food. Conventionally produced crops had a significantly higher content of N; organically produced crops had a significantly higher content of phosphorus (P) and higher titratable acidity. There was no evidence of further differences in nutritional quality of the remaining 8 of 11 crop nutrient categories. A subsequent systematic review (29), comprising 223 studies, came to a similar conclusion, although a further review and meta-analysis of 343 studies by Barański et al. (30) reported greater concentrations of several secondary compound micronutrients, notably polyphenols. A general challenge of synthesizing data from “organic vs. conventional” comparisons is that crops will have been grown in different geographical locations, and with multiple differences in nutrient inputs and other confounding factors.

A review by Montgomery and Bikle (31), starts to pull apart the controversy around organic vs. conventional, and suggests looking at the effect of specific farming practices on soil health and nutritional content, rather than attempting to attribute improvements in nutrient density to highly variable and complex systems.

Two systematic reviews have recently reported linkages between general agronomic approaches and tomato fruit micronutrient quality. In a meta-analysis on the effects of N supply on tomato yield, water use efficiency and fruit quality, Cheng et al. (32) assessed 1,096 data pairs from 76 publications. Under N supply rates sufficient for optimal yield, vitamin C increased by 19%, whereas lycopene decreased by 11% and nitrate content of fruit increased by 60%, compared to low N-input conditions. The second study was a meta-analysis on the effects of deficit irrigation on tomato quality (33). They assessed 2,369 data pairs, from 83 publications. Under deficit irrigation compared to full irrigation conditions, vitamin C increased by 14% and lycopene increased by 10%, whereas beta-carotene decreased by 11%. This was driven by a single study and the effect on beta-carotene was not seen when the study was excluded. However, the influence of deficit irrigation on nutritional quality was highly dependent on soil properties including texture and bulk density. For example, vitamin C improved more on course soils than medium textured soils whereas lycopene concentrations were larger on medium than course textured soils (33).

## Study aims

The review by Montgomery and Bikle (31), gives a broad overview of practices beyond defined systems that have been linked to observed changes in nutritional and phytochemical profiles across a range of crops and highlights the types of studies, potential mechanisms as well as the challenges of accounting for the factors affecting crop growth and health. Due to the complexity of different cropping systems and agronomic approaches, and a lack of explicit definitions of RA approaches, we adopted a wide literature search to ensure inclusion of papers on an agreed set of different RA practices. In contrast to the Bourne et al. (26) study, we considered practices intended to improve “soil health” and “landscape health” therefore included water management practices, rather than those which explicitly reported only soil health indicators.

We hypothesize that agronomic practices aligned to RA principles improve nutritional quality of edible portions of crops. The aim of this review was to identify where RA approaches can significantly improve crop micronutrient quality in the edible portion of field grown crops. We aimed to identify important food crops where there is robust evidence for positive nutritional effects linked to specific classes of practices. The study focused on a selection of mineral and secondary metabolites (vitamins) with well-established benefits to human health. It was guided by the following specific objectives:

1. To conduct a scoping review to generate the strength of evidence on effects of RA on crop nutrition.
2. To identify research gaps and weaknesses of current studies reporting on effects of RA and crop nutrition.
3. To encourage and recommend further research in RA and crop nutrition.

## Methods

To review the evidence on whether RA approaches can improve crop micronutrient quality, search terms were developed following discussions between co-authors, to define the scope of this study. We based the review on the Web of Science (Clarivate) data base, using the publication period of 2000–2021 (searches conducted in October 2021). We considered different RA approaches as “input” terms, using a range of keywords linked with the “or” Boolean operator. We adopted the same approach for “Crop Type,” based upon crops commonly consumed and used in food manufacturing. We considered crop micronutrient quality as an “output” term. These three terms were linked using the “AND” Boolean operator. The full search term string was:

(“conservation agricultur*” or “crop rotation” or “soil type” or “permaculture” or “agroforestry” or “agro-forestry” or “intercropping” or “inter-cropping” or “monoculture” or “mono-culture” or “agro-ecolog*” or “pixel farming” or “strip farming” or “soil microbial diversity” or “soil bacteria” or “soil fungi” or “mycorrhizal fungi” or “irrigation” or “fertigation” or “fertigation management” or “agroecolo*” or “manure” or “regenerative agricultur*” or “integrated soil fertility management” or “zero tillage” or “minimum tillage” or “soil organic matter” or “soil organic carbon”) AND (“wheat” or “tomato” or “carrot” or “rice” or “onion” or “lentils” or “pulses” or “beans” or “cereal*” or “grain legumes”) AND (“crop nutrient content” or “micronutrien*” or “vitami*” or “tocopherol” or “riboflavin” or “folate” or “zinc” or “iron” or “ferritin” or “magnesium” or “potassium” or “fibre” or “crop nutritional quality” or “selenium” or “calcium” or “beta carotene” or “ascorbic acid” or “iodine” or “mineral composition” or “trace elements”).

Truncated words (i.e., conservation agricultur*, agro-ecolog*, agroecolo*, agricultur*) were used to include words such as conservation agriculture, conservation agricultural practices, agro-ecology, agroecological region, agroecological zones, agroecologies, agriculture, agricultural practices, agricultural technologies, respectively. Articles were imported from Web of Science into Zotero (version 5.0.96.4; Roy Rosenzweig Center for History and New Media, 2016; www.zotero.org), using the RIS format which captured Author, Title, Source, Abstract and Meta Data (publication date, volume, and issue number, DOI, etc.). A wide literature search was based on three selection steps: (1) an initial search in which a set of RA-related agronomic practices and a crop micronutrient quality outcome was reported in the abstract or keywords of a paper in the Web of Science (Clarivate) data base, for a set of pre-determined crop types, for the period 2000–2021; (2) a manual review of all of the abstracts returned from this initial search, using the same criteria; (3) a manual assessment of the reported outcome of experimental studies conducted under “field” conditions, in which two or more treatment factors relevant to RA and a crop micronutrient quality measurement in the edible portions of the crop was reported. Each abstract was read by one of three co-authors (MGM-K, MRB and RML), with abstracts allocated according to the alphabetical position of the first author’s name on the paper to avoid crop-specific biases. Abstracts that were considered within the scope of an RA approach were then copied into a Zotero sub-folder according to primary crop-type: “alliums” (*Allium* spp.), “maize,” “other,” “pulses” (*Fabaceae* family), “rice” (*Oryza sativa* L.), “tomato,” “wheat.” Other studies were placed into various “excluded” sub-folders.

## Results

### Screening output

**Figure 2:**
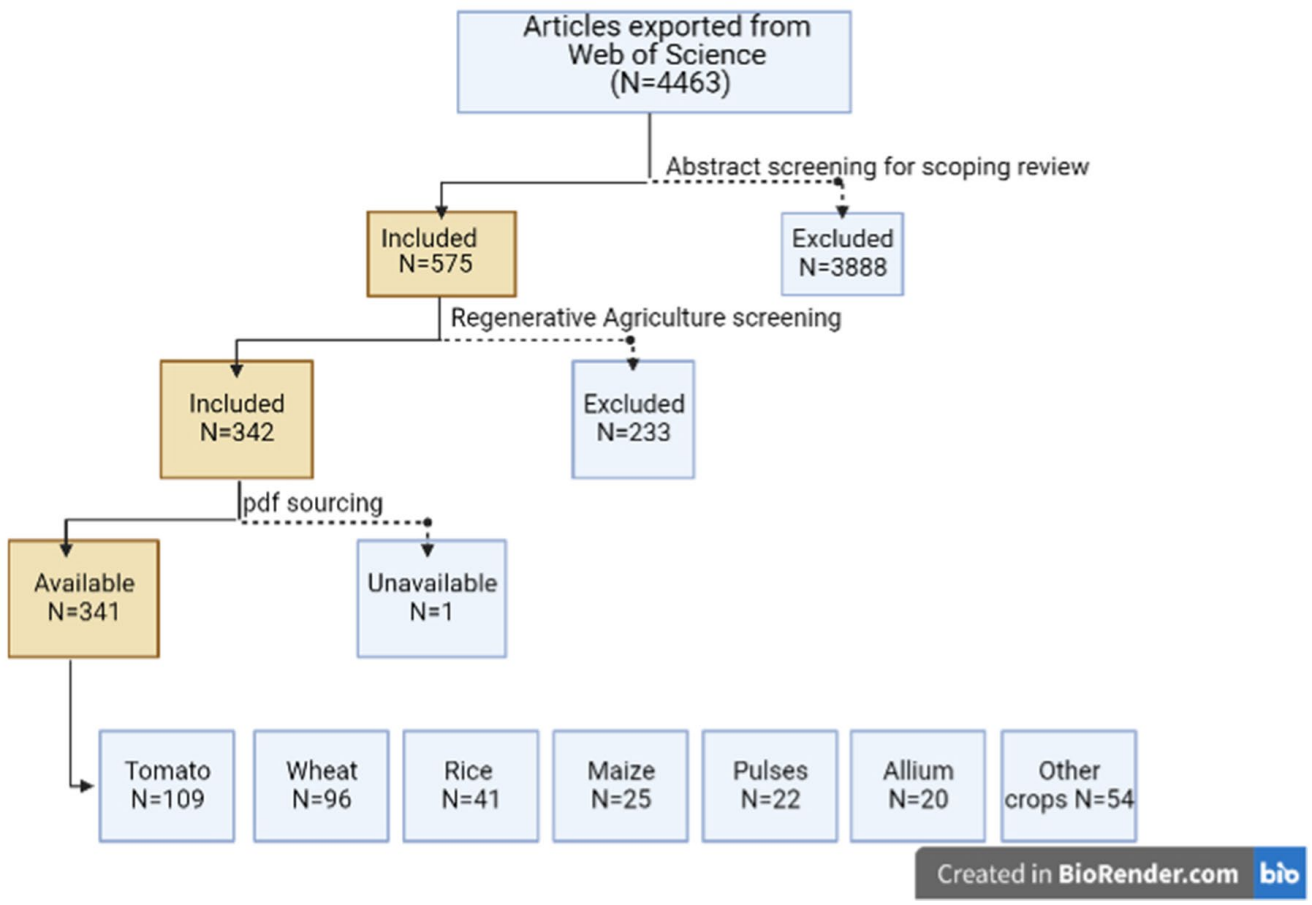
Flow chart on scoping review process. Dash line denotes exclusion. Created using BioRender (https://app.biorender.com/).

A total of 4,463 papers were returned from this search (Figure 2; https://www.zotero.org/groups/4466584/unilever_regenerative_agriculture/library). Whilst these search terms were not comprehensive in terms of RA approaches, crop types, or nutritional quality metrics, the sample size was considered sufficiently representative to enable a robust analysis of the evidence. The first (non-conservative) screen of RA approaches and crop micronutrient quality resulted in 575 abstracts being retained (Figure 2; https://www.zotero.org/groups/4500243/crop_type_screening_for_unilever_regenerative_agriculture). These abstracts were then subjected to a second round of review (MGM-K and MRB). During this second round, we removed abstracts reporting non-RA approaches such as agronomic biofortification (see Discussion), fertigation, liming, and the use of contaminated/saline wastewaters and sewage sludges as the primary objective of the study, also studies whose primary focus was to reduce the transfers of contaminants (e.g., heavy metals) into crops. For tomato, we removed abstracts in which crops had been grown in hydroponics or other soil-less systems. This second round of review reduced the number of abstracts to 342 for further analysis; 341 of these were secured in full-paper .pdf format from library subscriptions or inter-library loans. Where data for more than one crop type was reported in a study, the study was copied across multiple crop sub-folders in Zotero, providing a total of 367 records.^2^ The 367 records were combinations of RA agronomic approaches [“Organic Inputs” (including animal and green manures, cover crops, crop rotations, crop residues, composts, biochars; excluding those studies whose primary focus was on contaminated soils or amendments), “Tillage,” “Intercropping,” “Biostimulants” (e.g., AMF; plant growth promoting bacteria, PGPB), and “Irrigation” (typically deficit-irrigation systems)]. The records were then allocated as: “tomato” (*n* = 109), “wheat” (*n* = 96), “rice” (*n* = 41), “maize” (*n* = 25), “pulses” (*n* = 22), “alliums” (*n* = 20), and “other” crop types (30 types, representing 54 studies (Figure 2).

A full review of these 367 records was conducted (EJMJ, MGM-K and MRB). We retained the same inclusion criteria and categorized the type of RA approach adopted as: “Organic Inputs” (including animal and green manures, cover crops, crop rotations, crop residues, composts, biochars; excluding those studies whose primary focus was on contaminated soils or amendments), “Intercropping,” “Tillage,” “Biostimulants” (e.g., AMF, PGPRs), and “Irrigation” (typically deficit-irrigation systems for horticultural crops; excluding studies whose primary focus was on contaminated/toxic/saline/wastewater irrigation). Studies whose primary focus was on experimental interventions outside of these five categories were excluded. Studies conducted in the field were included; studies conducted in pots or containers were excluded. For tomato, protected field crops were included when conducted in open soil environments. Studies reporting micronutrient concentrations in crop edible tissue were included; studies reporting micronutrient concentrations only in non-edible shoot tissues were excluded. Where possible, RA approaches were compared to “conventional” treatments which typically involved recommended applications of NP and potassium (K) mineral fertilizer. Studies were excluded if a statistical analyses of a “control” versus an “RA” input condition was not reported explicitly. Reviews and surveys were excluded, as were studies published in a language other than English.

The evidence for an effect of RA approach on the micronutrient concentration of the edible portion of the crop was recorded, for each micronutrient reported within a study, as: (1) evidence of a statistical significant increase (↑) or decrease (↓) in the micronutrient concentration of the crop, under one or more of the RA/control contrasts reported in the study; (2) no evidence of a statistical significant change in the micronutrient concentration of the crop, in any of the RA conditions reported in the study (↔); (3) evidence of both a statistical significant increase and a statistical significant decrease in the micronutrient concentration of the crop, under one or more RA conditions reported in the study (↕). If a study reported a statistical significant increase or decrease in the micronutrient concentration of the crop in a subset of treatment levels, or years, and no statistical significant changes in another subset of years or treatment levels, then this was recorded as evidence of a significant increase (↑) or decrease (↓).

Results are reported in order of numbers of studies reviewed in full for each crop type, i.e., tomato, wheat, rice, maize, pulses, alliums, and other crops.

### Tomato

A total of 109 papers on tomato were read in full. There were 51 studies whose primary focus was on Organic Inputs; 35 studies on Irrigation; 10 studies on Biostimulants; 2 studies on Intercropping. Details of the 109 studies are given in Supplementary Table 1. The micronutrient with the greatest coverage among tomato studies is vitamin C/ascorbic acid, which was reported in 74 of the papers, followed by lycopene (*n* = 34), beta-carotene/carotenoids (*n* = 21), polyphenols/phenolics (*n* = 3), flavonoids (*n* = 2), and tocopherols (n = 2). Fewer tomato studies reported mineral micronutrient concentrations compared to vitamins and secondary nutrient compounds: Ca (n = 16), Mg (n = 12), Fe (*n* = 8), Zn (*n* = 9), Cu (*n* = 7), Mn (*n* = 7), Se (*n* = 1).

Fifty-two of the 109 studies on tomato were excluded from this scoping review: 28 pot studies (i.e., closed soil systems) which comprised: 11 Organic Inputs studies; 8 Irrigation studies; 6 Biostimulant studies; 2 non-RA studies. From tomato studies conducted in open soil (“field”) conditions, which could be either protected (e.g., polytunnel) or non-protected systems, 10 papers were excluded due to being non-RA which comprised: 3 Irrigation studies based on physical interventions (e.g., installation of improved drainage systems); 3 conventional fertilizer studies; 2 wastewater studies; 1 seedling (pre-transplant) study; 1 study which used compositing to increase atmospheric carbon dioxide (CO_2_) in a protected field system. A further 4 field studies were excluded due to no fruit micronutrients being reported (e.g., (34) and Asri (35), reported leaf/shoot micronutrients). A further 10 exclusions comprised: 2 studies compared organic and non-organic product batches, confounded by location; 4 review articles; 4 non-English language papers. Of the 57 included studies, Organic Inputs (*n* = 29), Irrigation (*n* = 23), Biostimulants (*n* = 3), Intercropping (*n* = 2), were represented among the RA approaches (Table 1).

**Table 1:** Studies reporting effects of regenerative agriculture on vitamin C concentration in tomato.

Primary regenerative ag. strategy reported	Micronutrient [evidence of direction of change, ↔,↑,↓,↕]	Number in included studies	References
Organic Inputs	Vitamin C [increase, ↑]	11	Yu et al. (36), Abduli et al. (37), Song et al. (38), Wang et al. (39), Dinu et al. (40), Özer (41), Jin et al. (42), Duan et al. (43), Zhang et al. (44), Guo et al. (45), Nabaei et al. (46)
	Vitamin C [decrease, ↓]	2	Petropoulos et al. (47), Huang et al. (48)
	Vitamin C [no significant change, ↔]	12	Tuzel et al. (49), Ece and Uysal (50), Polat et al. (51), Rady (52), Ceglie et al. (53), Majkowska-Gadomska (54), She et al. (55), Mukherjee et al. (56), Qahraman et al. (57), Rosa-Martinez et al. (58), Wu et al. (59), Turhan and Ozmen (60)
	Vitamin C [increases and decreases, depending on treatment, ↕]	0	n.a
Irrigation	Vitamin C [increase, ↑]	13	Chen et al. (61), Helyes et al. (62), Shao et al. (63), Abdel-Razzak et al. (64), Nangare et al. (65), Du et al. (66), Guida et al. (67), Wang et al. (68), Marti et al. (69), Cui et al. (70), Samui et al. (71), Al-Selwey et al. (72), Wu et al. (73)
	Vitamin C [decrease, ↓]	2	Helyes et al. (74), Turhan et al. (75)
	Vitamin C [no significant change, ↔]	2	Helyes et al. (76), Al-Harbi et al. (77)
	Vitamin C [increases and decreases, depending on treatment, ↕]	0	n.a
Biostimulants	Vitamin C [increase, ↑]	2	Tiyagi et al. (78), Le et al. (79)
	Vitamin C [decrease, ↓]	0	n.a
	vitamin C [no significant change, ↔]	0	n.a
	Vitamin C [increases and decreases, depending on treatment, ↕]	0	n.a
Intercropping	Vitamin C [increase, ↑]	1	Liu et al. (80), (with garlic)
	Vitamin C [decrease, ↓]	0	n.a
	Vitamin C [no significant change, ↔]	0	n.a
	Vitamin C [increases and decreases, depending on treatment, ↕]	1	Demir and Polat (81) (with lettuce)

n.a. = not applicable.

Among the studies on tomato whose primary focus was Organic Inputs, there was some evidence that fruit vitamin C concentrations increased (Table 1; also see Supplementary Table 1); 11 studies reported an increase, 12 studies reported no significant change, and 2 studies reported a decrease. Among the (deficit) Irrigation studies on tomato, there was strong evidence that fruit vitamin C concentrations increased: 13 studies reported an increase, 2 studies reported no significant change, and 2 studies reported a decrease in fruit vitamin C concentrations. Among the studies on biostimulants (using PGPBs) on tomato, 2 studies reported an increase, and no studies reported a decrease, or a non-significant change in fruit vitamin C concentrations. Among the Intercropping studies on tomato, 1 study reported an increase, and 1 study reported significant increases and decreases in fruit vitamin C concentrations within the same study (81).

For fruit lycopene, 3 Organic Inputs studies reported an increase in concentrations; 3 studies reported no significant changes; 1 study reported a decrease (Table 2). Among Irrigation studies on tomato, 7 reported an increase in fruit lycopene concentrations; 4 studies reported no significant changes; 1 study reported a decrease; 1 study reported significant increases and decreases in fruit lycopene concentrations within the same study. Among the Biostimulants studies (PGPBs) on tomato, 1 reported both significant increases and decreases in fruit lycopene concentrations within the same study (79).

**Table 2:** Studies reporting effects of regenerative agriculture on carotenoids, flavonoids, lycopene, and phenolics contents in tomato.

Primary regenerative ag. strategy reported	Carotenoids, *Flavonoids, Lycopene and Phenolics [evidence of direction of change, ↔,↑,↓,↕]	Number in included studies	References
Organic Inputs	Carotenoids [increase, ↑]	2	Dinu et al. (40), Turhan and Ozmen (60)
	Carotenoids [decrease, ↓]	1	Ceglie et al. (53)
	Carotenoids [no significant change, ↔]	1	Rosa-Martinez et al. (59)
	Carotenoids [increases and decreases, depending on treatment, ↕]	0	n.a
		
	Lycopene [increase, ↑]	3	Wang et al. (39), Mukherjee et al. (56), Turham and Ozmen (68)
	Lycopene [decrease, ↓]	1	Petropoulos et al. (47)
	Lycopene [no significant change, ↔]	3	Wu et al. (58), Huang et al. (48), Rosa-Martinez et al. (59)
	Lycopene [increases and decreases, depending on treatment, ↕]	0	n.a
Irrigation	Carotenoids [increase, ↑]	1	
	Carotenoids [decrease, ↓]	0	n.a
	Carotenoids [no significant change, ↔]	1	
	Carotenoids [increases and decreases, depending on treatment, ↕]	0	n.a
		
	Lycopene [increase, ↑]	7	Helyes et al. (82), Helyes et al. (62), Helyes et al. (83), Pék et al. (84), Turhan et al. (75), Du et al. (66), Samui et al. (71)
	Lycopene [decrease, ↓]	1	Liu et al. (85)
	Lycopene [no significant change, ↔]	4	Helyes et al. (74, 76), Martí et al. (56), Wu et al. (73)
	Lycopene [increases and decreases, depending on treatment, ↕]	1	Wang et al. (68)
		
	Phenolics [increase, ↑]	4	Helyes et al. (74), Helyes et al. (62), Helyes et al. (83), Pék et al. (84)
	Phenolics [decrease, ↓]	0	n.a
	Phenolics [no significant change, ↔]	0	n.a
	Phenolics [increases and decreases, depending on treatment, ↕]	0	n.a
Biostimulants	Carotenoids [increase, ↑]	0	n.a
	Carotenoids [decrease, ↓]	0	n.a
	Carotenoids [no significant change, ↔]	0	n.a
	Carotenoids [increases and decreases, depending on treatment, ↕]	1	Le et al. (79)
		
	Lycopene [increase, ↑]	0	n.a
	Lycopene [decrease, ↓]	0	n.a
	Lycopene [no significant change, ↔]	0	n.a
	Lycopene [increases and decreases, depending on treatment, ↕]	1	Le et al. (79)

* Increase [↑] in flavonoids reported in one irrigation study (83). n.a. = not applicable.

For fruit beta-caroteine/carotenoids, 3 Organic Inputs studies reported an increase in concentrations; 1 study reported no significant changes; 2 studies reported a decrease (Table 2). Among the Irrigation studies on tomato, 1 study reported an increase in fruit beta-caroteine/carotenoids concentrations; 4 studies reported no significant changes; no studies reported a decrease. Among the Biostimulants studies (PGPBs) on tomato, 1 reported significant increases and decreases in fruit beta-caroteine/carotenoids concentrations within the same study (79). For fruit tocopherols and polyphenols/phenolics, there was one study under Organic Inputs, for each nutrient, that reported an increase in fruit concentrations. Among the Irrigation studies on tomato, 1 reported significant increases and decreases in fruit tocopherol concentrations within the same study (74); 4 studies reported increases in fruit polyphenols/phenolics concentrations; 1 study reported increases in fruit flavonoid concentrations (83).

There were many fewer studies on tomato fruit mineral micronutrient concentrations under RA approaches than for secondary micronutrients, all of which were in the Organic Inputs category. For fruit Fe and Zn concentrations, 2 studies reported an increase (51, 86); 1 study reported no significant changes; no studies reported a decrease in either micronutrient. For fruit Ca concentration, 2 studies reported an increase; 2 studies reported no significant changes; 1 study reported a decrease. For fruit Mg concentration, increases were reported in 1 study; 2 studies reported no significant changes; no studies reported a decrease. There was 1 study which reported an increase in fruit Se concentration (48), and one study which reported an increase in fruit copper (Cu) concentration (51).

### Wheat

A total of 96 papers on wheat were read. There were 43 studies whose primary focus was on Organic Inputs; 10 studies on Biostimulants; 5 studies on Tillage; 1 study on Intercropping; and 6 Surveys. There were 4 studies reporting other non-RA techniques. Details of the 96 reviewed studies are given in Supplementary Table 2.

Fifty-two of these 96 studies were excluded from this scoping review: 13 pot studies; 6 survey studies; 4 non-RA field studies; 19 studies in which only micronutrient concentration data for non-edible portions were reported; 2 studies not in English language; 1 conference abstract; 1 review paper; 2 studies with no quantitative data; 1 study with no comparator for the RA treatment; 1 study not on wheat; 2 studies with potassium data only. Of the 44 included studies, Organic Inputs (*n* = 34), Biostimulants (*n* = 6), Intercropping (*n* = 1), and Tillage (*n* = 5) were represented among the RA approaches (Supplementary Table 2), noting that Woźniak (87) tested Organic Inputs and Tillage treatments while Shivay et al. (88) tested Organic Inputs and Biostimulants approaches.

Among the 34 studies on wheat whose primary focus was Organic Inputs and reporting grain mineral concentrations, there was some evidence for increases in grain micronutrients. For Zn, 11 studies reported increases in grain Zn concentration; 3 studies reported decreases in grain Zn concentration; 8 studies did not report a significant change in grain Zn concentration; 1 study reported significant increases and decreases in grain Zn concentration (89). Increases in grain Zn concentration were attributed to co-application of Organic Inputs with crop residues and mineral N fertilizer [i.e., urea-CH_4_N_2_O; e.g., (90, 91)]. Decreases in grain Zn concentration were reported when organic farming management followed grass/clover as a pre-crop (92) and under long term biochar application (93). Gondek (89) reported an increase in grain Zn concentration of wheat when sewage sludge was applied and a decrease in grain Zn concentration when swine farmyard manure and compost from plant and biodegradable waste were applied in the 3^rd^ year of experimentation. For Fe, 4 studies reported increases in grain Fe concentration; 2 studies reported decreases in grain Fe concentration; 12 studies did not report a significant change in grain Fe concentration. For Mg, 2 studies reported increases in grain Mg concentration; 2 studies reported decreases in grain Mg concentrations; 4 studies did not report a significant change in grain Mg concentration. For Ca, 1 study reported increases in grain Ca concentrations; no studies reported decreases in grain Ca concentration; 5 studies did not report a test of significance or reported no significant changes in grain Ca concentration.

Among the 6 studies on wheat whose primary focus was Tillage, there was no evidence for changes in grain Zn and Fe concentrations. For Zn, 0 studies reported increases in grain Zn concentrations; 0 studies reported decreases in grain Zn concentrations; 2 studies did not report a test of significance or reported no significant changes in grain Zn concentration; and 1 study reported significant increases and decreases in grain Zn concentration (87). For Fe, 0 studies reported increases in grain Fe concentrations; 0 studies reported decreases in grain Fe concentrations; 4 studies did not report a test of significance or reported no significant changes in grain Zn concentration.

Among the 6 studies on wheat whose primary focus was Biostimulants, there was some evidence for increases in grain Zn concentrations but mixed evidence for Fe. For Zn, 4 studies reported increases in grain Zn concentrations; 0 studies reported decreases in grain Zn concentrations; 2 studies did not report a test of significance or reported no significant changes in grain Zn concentration. For Fe, 2 studies reported increases in grain Fe concentration; 1 study reported decreases in grain Fe concentration; 1 study did not report a test of significance or reported no significant changes in grain Fe concentration.

### Rice

A total of 41 papers on rice were read. There were 25 studies whose primary focus was on Organic Inputs; 9 studies on Irrigation; 2 studies on Biostimulants; 1 study on Tillage; 2 Surveys. There were 2 studies reporting other non-RA techniques. Details of the 41 reviewed studies are given in Supplementary Table 3.

18 of these 41 studies were excluded from this scoping review: 7 pot studies; 2 surveys; 3 non-RA field studies; 3 field studies in which rice grain micronutrient concentration data were not reported; 3 field studies in which only micronutrient concentration data for non-edible portions of rice were reported. Of the 23 included studies for rice, Organic Inputs (*n* = 16) and Irrigation (*n* = 7) were represented among the RA approaches (Supplementary Table 3).

Among the 16 studies on rice whose primary focus was Organic Inputs, there was strong evidence for increases in grain Zn and Fe concentration. For Zn, 14 studies reported increases in grain Zn concentration; 1 study reported no significant changes in grain Zn concentration (94); 1 study reported significant increases and decreases in grain Zn concentration (95). For Fe, 5 studies reported increases in grain Fe concentration; 2 studies reported decreases in grain Fe concentration; 3 studies reported significant increases and decreases in grain Fe concentration [e.g. (96), who also reported increases and decreases in grain Ca and Mg concentration under different treatments].

Among the 7 studies on rice whose primary focus was Irrigation (deficit techniques, including alternate wetting and drying, AWD), the results were less conclusive than for studies on Organic Inputs. For Zn, 3 studies reported increases in grain Zn concentration; 2 studies reported decreases in grain Zn concentration; 1 study reported no significant changes in grain Zn concentration (97). For Fe, 3 studies reported decreases in grain Fe concentration, and none reported an increase. For Se, 1 study reported an increase in grain Se concentration (98) and 1 study reported a decrease in grain Se concentration (99).

### Maize

A total of 25 papers on maize were read. There were 11 studies whose primary focus was on Organic Inputs; 6 studies on Intercropping; 2 studies on Tillage; 1 Survey. There were 5 studies reporting other non-RA techniques. Details of the 25 reviewed studies are given in Supplementary Table 4.

Fifteen of these 25 studies were excluded from this scoping review: 5 pot studies; 1 survey study; 4 non-RA field studies; 2 field study in which micronutrient concentration data were not reported; 3 field studies in which only micronutrient concentration data for non-edible portions were reported. Of the 10 included studies, Organic Inputs (*n* = 4), Intercropping (*n* = 5), and Tillage (*n* = 1) were represented among the RA approaches (Supplementary Table 4).

Among the 4 included studies on maize whose primary focus was Organic Inputs, there was evidence for significant increases in mineral and vitamin micronutrient concentration (Supplementary Table 4). For Fe and Zn, all 4 studies reported increases in grain concentration. One of these studies also reported an increase in grain Ca and Mg concentrations (100). Another of these studies, on baby sweetcorn, reported an increase in vitamin C concentration (101).

Among the 5 included studies on maize whose primary focus was Intercropping, two studies reported an increase (102–104) and one study reported a decrease (105) in grain Fe concentration. One study reported an increase (102, 103), and two studies reported a decrease (102, 103, 105), in grain Zn concentration. One study reported an increase in grain Ca concentration and a decrease in grain Mg concentration under intercropping (106). In the Tillage study, there was no significant change in grain Zn concentration (107).

### Pulses

Of the 22 studies read which included a pulse crop, there were 7 studies on *Phaseolus vulgaris* (common bean, snap bean, pinto bean, green/yellow bean), 5 studies on *Arachis hypogea* (peanut, groundnut), 3 studies on *Pisum sativum* (pea), 2 studies on *Vigna unguiculata* (cowpea), and 2 studies on *Vicia faba* (faba bean). There were 12 studies whose primary focus was on Organic Inputs; 7 studies on Intercropping; 1 study on Biostimulants; 1 study on Tillage; 1 Survey. Details of these 22 reviewed studies are given in Supplementary Table 5.

Fifteen of these studies were excluded from this scoping review: 4 pot studies; 1 review; 1 variety trial (cowpea) on reduced tillage; 1 field study with no control comparison; 1 field study in which micronutrient concentration data were not reported; 7 field studies in which only micronutrient concentration data for non-edible portions were reported. Of the 7 included studies, Organic Inputs, Intercropping, and Biostimulants were represented among the RA approaches (Supplementary Table 5).

Among the 5 included studies on pulse crops whose primary focus was Organic Inputs (Supplementary Table 5), 2 studies reported an increase in mineral micronutrient concentration (copper-Cu; Fe; manganese-Mn; Zn), whereas 3 studies reported no significant changes for the concentration of these mineral micronutrients; one of these studies also reported no significant changes for vitamin C concentration. There was no evidence of decreased concentration of micronutrients in seeds of pulses under Organic Inputs. The 1 pulse study on Intercropping (pea with oat; *Avena sativa*) that was included reported no significant changes in seed Ca or Mg concentration. The 1 pulse study whose primary focus was Biostimulants (AMF inoculations; Supplementary Table 5) reported an increase in vitamin C concentration.

### Alliums

Of the 20 studies read which included an *Allium* crop, there were 13 studies on onion, 3 studies on garlic, 2 studies on leek, 1 study on shallots, and 1 study which reported micronutrient data for both onion and garlic. There were 14 studies whose primary focus was on Organic Inputs; 2 studies on Biostimulants; 1 study on Intercropping; 1 study on Irrigation; 1 study on Fertigation; 1 Survey. Details of these 20 reviewed studies are given in Supplementary Table 6.

Eight of these 20 studies were excluded from this scoping review: 1 pot study; 1 survey study; 1 fertigation study; 2 field studies in which micronutrient concentration data were not reported; 1 field study in which only micronutrient concentration data for non-edible portions were reported; 1 field study lacking a non-regenerative control; 1 non-English language study. Of the 12 included studies, only Organic Inputs and Biostimulants were represented among the RA approaches (Supplementary Table 6).

Among the 10 included studies on *Allium* crops whose primary focus was Organic Inputs (Supplementary Table 6), three studies reported an increase in vitamin C concentration in their edible portions, whereas two studies reported no significant changes. One *Allium* crop study reported an increase in mineral micronutrient concentrations, but five studies reported no significant changes and two reported a decrease in mineral micronutrient concentrations. One *Allium* crop study reported an increase in flavonoid and phenolic concentrations, and one reported no significant changes. Among the three included studies on *Allium* crops whose primary focus was Biostimulants (AMF inoculations; Supplementary Table 6), one study reported an increase in vitamin C concentration and two studies reported no significant changes. All three *Allium* crop studies whose primary focus was Biostimulants (AMF) reported an increase in one or more mineral micronutrient concentrations.

### Other crops

Of the 54 papers read which included ‘other’, crop types, the most common horticultural crop types were carrot (*Daucus carota*; *n* = 11), lettuce (*Lactuca sativa*; *n* = 7), pepper (*Capsicum annuum*; *n* = 6), cabbage (*Brassica oleracea* var. *capitata*; *n* = 5), and potato (*Solanum tuberosum*; *n* = 4). The most common cereal crop types were barley (*Hordeum vulgare*; *n* = 4), then sorghum (*Sorghum bicolor*; *n* = 3) and pearl millet (*Pennisetum glaucum*; *n* = 3). The number of species in this category are given in Table 3; these add to 69 studies because some of the 54 papers included multiple crop types. There were 44 studies whose primary focus was on Organic Inputs; 14 studies on Intercropping; 6 studies on Biostimulants; 3 studies on Irrigation; 2 studies on Fertigation. Details of these 69 reviewed studies are given in Supplementary Table 7.

**Table 3:** Species of other crops represented in studies included in the scoping review.

Species	Number of studies
Cereal crops	
Barley (*Hordeum vulgare*)	4
Oat (*Avena sativa*)	2
Pearl millet (*Pennisetum glaucum*)	3*
Sorghum (*Sorghum bicolor*)	3
Non-cereal crops	
*Acacia mearnsii*	1
Broccoli (*Brassica oleracea* var. *italica*)	1
Brussels sprout (*Brassica oleracea var. gemmifera*)	1
Cabbage (*Brassica oleracea var. capitata*)	5
Carrot (*Daucus carota*)	11
Cauliflower (*Brassica oleracea var. botrytis*)	3
Cocoyam (*Colocasia esculent*)	1
Cucumber (*Cucumis sativus*)	1
Elephant foot yam (*Amorphophallus paeoniifolius*)	1
Ice plant (*Mesembryanthemum crystallinum*)	1
Jute mallow (*Corchorus olitorius*)	1
Korean ginseng (*Panax ginseng*)	1
Lettuce (*Lactuca sativa*)	7
Melon (*Cucumis melo*)	1
Mulberry (*Morus alba*)	1
Mustard (*Sinapis alba*)	1
Oilseed rape (*Brassica napus*)	2
Pepper (*Capsicum annuum*)	5
Potato (*Solanum tuberosum*)	4
Red clover (*Trifolium pratense*)	1
Squash (*Cucurbita*)	1
Strawberry (*Fragaria* × *ananassa*)	1
Sunflower (*Helianthus annuus*)	1
Sweet cherry (*Prunus avium*)	1
Sweet potato (*Ipomoea batatas*)	1
Various species (one temporal survey and a review)	2

* Pearl millet counted twice in Bana et al. (108), under organic inputs and intercropping.

Thirty-nine of the 69 studies were excluded from this scoping review: 7 non-RA studies; 17 pot studies; 2 field studies in which micronutrient data were not reported; 8 field studies in which only micronutrient concentration data for non-edible portions were reported; 2 survey studies; 1 review paper; 1 non-English language study; 1 study could not be accessed through library (109). Of the 30 included studies, Organic Inputs (*n* = 21), Intercropping (*n* = 6), Irrigation (*n* = 2), and Biostimulants (*n* = 1) were represented among the RA approaches (Supplementary Table 7).

Among the 21 included studies on Other Crops whose primary focus was Organic Inputs, there was little evidence for significant changes in mineral or vitamin micronutrient concentration (Supplementary Table 7). For Fe and Zn, 8 studies reported no significant changes in concentration. One study (pearl millet) reported an increase in Fe and Zn concentration (108) and 1 study (pepper) reported an increase in Zn concentration (110). One study (barley) reported a decrease in Fe concentration (111). For Ca and Mg, there were no increases in concentration in 13 and 11 studies, respectively; 1 of these Ca studies (carrot) reported a decrease in Ca concentration (112). For vitamin C, there were no significant changes in concentration in 6 studies; 1 study (strawberry) reported an increase in concentration (113). For beta-carotene, there were no significant changes in concentration in 2 studies.

Among the 6 included studies on Other Crops whose primary focus was Intercropping, 1 study (pearl millet) reported an increase in Fe and Zn concentration (108); 1 study (cabbage) reported no significant changes in Fe concentration (114). For Ca and Mg, 1 study (carrot) reported an increase in concentration (115); 1 study (oat) reported a decrease in concentration (116) and 1 study (cabbage) reported no significant changes in concentration (114). For vitamin C, one study (lettuce) reported significant increases and decreases in concentration within the Intercropping study (81). The two included Irrigation studies on Other Crops, whose primary focus was Intercropping, had no effect on carotenoid concentration. The one included Biostimulant study (potato) showed an increase in vitamin C concentration.

### Summary of results by regenerative agriculture practice and crop type

To summarize the results from this scoping review, we focused on Zn, Fe, and vitamin C, as examples of mineral and secondary micronutrients with the greatest coverage. For each combination of crop and RA category, we considered the strength of the evidence on the following arbitrary categories based on the proportion of studies that showed a significant increase (or decrease) in micronutrient content in a RA treatment vs. control: >66% = “Good evidence”; 33–66% = “Some evidence”; <33% = “Little/no evidence.” This evidence is summarized and presented for any increase and/ or decrease in Zn, Fe and vitamin C in all crop types in Table 4. Percent change values (evidence of increase or decrease in a nutrient) for different crops was calculated by dividing the total number of studies reporting a significant increase (or decrease) in a particular nutrient by the total number of studies reporting the nutrient within a particular RA approach (i.e., Organic Inputs). This value was then multiplied by 100 (Excel Supplementary file).

**Table 4:** Percentage of studies reporting increases (and decreases) in zinc, iron and vitamin C with respect to a specific Regenerative Agriculture practice.

	**Regenerative practice**	**i. Evidence of increase (%)**							**ii. Evidence of decrease (%)**						
		Wheat	Rice	Tomatoes	Alliums	Pulses	Maize	Other crops	Wheat	Rice	Tomatoes	Alliums	Pulses	Maize	Other crops
Zinc															
	Organic inputs	48 (12/25)	94 (15/16)	67* (2/3)	20* (1/5)	40* (2/5)	100* (4/4)	18 (2/11)	16 (4/25)	6 (1/16)	0	0	0	0	0
	Irrigation		50 (3/6)							33 (2/6)					
	Biostimulants	67 (4/6)			33* (1/3)				0			0			
	Intercropping	0					33* (1/3)	100* (1/1)	0					67* (2/3)	0
	Zero tillage	33* (1/3)					0		33* (1/3)					0	
Iron															
		Wheat	Rice	Tomatoes	Alliums	Pulses	Maize	Other crops	Wheat	Rice	Tomatoes	Alliums	Pulses	Maize	Other crops
	Organic inputs	22 (4/18)	80 (8/10)	67* (2/3)	20* (1/5)	33* (1/3)	100* (4/4)	10 (1/10)	11 (2/18)	50 (5/10)	0	20 (1/5)	0	0	10 (1/10)
	Irrigation		0							100* (3/3)					
	Biostimulants	60* (3/5)			67* (2/3)				20* (1/5)			0			
	Intercropping	100* (1/1)					67* (2/3)	50* (1/2)	0					33* (1/3)	0
	Zero tillage	0							0						
Vit. C															
		Wheat	Rice	Tomatoes	Alliums	Pulses	Maize	Other crops	Wheat	Rice	Tomatoes	Alliums	Pulses	Maize	Other crops
	Organic inputs	100* (1/1)		44 (11/25)	60* (2/5)			14 (1/7)			8 (2/25)				
	Irrigation			76 (13/17)							12 (2/17)				
	Biostimulants			100* (2/2)	33* (1/3)	100* (1/1)		100* (1/1)							
	Intercropping			100* (2/2)				50* (1/2)			50* (1/2)				50* (1/2)
	Zero tillage														

**Key:**

1. Vit. C = Vitamin C.

2. Orange = Some evidence/inconclusive (33-66%).

3. Blue = Good evidence for positive effects (>66%).

4. Gray = Little/No evidence (<33%).

5. Numbers in parenthesis denote the number of studies reporting an increase/decrease over the total number of studies reporting the nutrient within a specific Regenerative Agriculture (RA) practice.

6. * denotes small number of studies (i.e., <6).

7. Blank = Significant research gap.

8. Cells are blank where no data were identified for the nutrient-RA practice combination.

9. Values are “0” where studies reported no increase or decrease in nutrient concentration.

10. Figures are percentages of studies showing a significant increase and/or decrease in the nutrient. Percentages may sum to >100 because some studies reported significant increases and decreases for the same nutrient-RA practice combination, e.g. at different sites, or for different specific treatments.

%change=Number of studies reportingasignificant increase or decrease inanutrientTotal number of studies reporting the nutrient withinaparticularRAapproachx100.

### Evidence of increases in the micronutrient concentrations of crop edible portions under different categories of RA practices

#### Organic Inputs

All crop types were represented in this category of RA practice, which comprised a wide range of organic inputs including the use of animal and green manures, crop rotations, cover crops, and various composts; typically compared to a control of conventional mineral fertilizer inputs. There is good or some evidence that the use of organic inputs increases Zn concentration in the grains/seeds of most field crops (i.e., rice = 94% of studies, wheat = 48%, maize = 100%, pulses = 40%; Table 4). Grain Fe concentration increased in 80% of rice studies, 22% of wheat studies, 100% of maize studies and 33% of studies on pulses. Similarly, some evidence of organic inputs effect on increasing Zn and Fe concentration in tomato was reported (67%). There is little/no evidence that organic inputs increase Zn concentration in the edible portions of alliums (20%), or other crops (18%). Similar evidence was reported for Fe concentration in alliums (20%) and other crops (10%). There is little/no evidence of organic inputs decreasing Zn or Fe with the exception of rice where a decrease in grain Fe concentration was reported in 50% of the studies, some of which focused on effects of poultry and vermicompost and 17-years continuous application of pig manure and straw on rice grain Fe concentration. There is some evidence that the use of organic inputs increases vitamin C concentration in the edible portions of wheat grown in rotation with a legume (100%, *n* = 1 study), tomato grown with biochar, mulch and vermicompost (44%) and alliums grown with green and animal manure (60%), with little/no evidence reported in other crops (14%). There is little/no evidence of any decreases in the vitamin C concentration in tomato or other crop types, noting there are few studies reporting vitamin C concentration under organic inputs for other crop types.

#### Irrigation

Rice, tomato, and other crops were represented in this category, which generally focused on reduced/deficit irrigation and alternate wetting and drying (AWD) compared to conventional practices. Altered irrigation is likely to have large effects on soil nutrient availability due to changes in redox conditions and associated changes in pH and other geochemical properties. There is some evidence that alternative irrigation strategies increased (50%) or decreased (33%) the Zn concentration in the edible portion of rice, which was the only crop represented in this category (Table 4). In contrast, good evidence of decrease in grain Fe concentration (100%) was reported. This was based on findings from three studies reporting grain Fe concentration in rice grown under alternative irrigation strategies. There is good evidence that alternative irrigation strategies increase the fruit concentration of vitamin C in tomato (76%), which was the only crop represented in this category. There is little/no evidence that alternative irrigation practices decrease the fruit concentration of vitamin C concentration in tomato (12%).

#### Tillage

Wheat and maize were the only crops represented in this category, which focused on reduced tillage practices compared to a conventionally tilled control. There is some evidence that alternative tillage strategies employed over 2 years, increased Zn concentration in the grains of wheat (33%) with little/no evidence reported in maize (0%; Table 4) grown under a zero-tilled field over a 17-year period. Similarly, there was some evidence that alternative tillage strategies decreased Zn concentration in the grains of wheat (33%) with no evidence reported in maize (0%). Contrary to tillage effects on Zn, no evidence of such effects was reported in all four studies measuring grain Fe concentration in wheat (evidence of 0%). None of the tillage studies reported grain vitamin C concentrations.

#### Biostimulants

All crops, except for rice and maize, were represented in this category of RA practices, which typically comprised AMF, PGPBs, bacterial strains (*Bacillus*, *Pseudomonas*, *Arthrobacter*, and *Azotobacter* species) and amino acids, although the number of studies captured in this review are small. There is some evidence that biostimulants increased Zn concentration in the edible portions of wheat (67%) and alliums (33%) with similar evidence of 60 and 67% increase in Fe concentration reported in wheat and alliums, respectively (Table 4). A 20% evidence of decrease in wheat grain Fe concentration with use of biostimulants was reported. No studies measured the effect of biostimulants on Zn or Fe concentrations in the edible portions of tomato, pulses, or other crops. There is good evidence that biostimulants increased the concentration of vitamin C in the edible portions of tomato (100%), pulses (100%), and other crops (potato; 100%), with some evidence of this effect in alliums (33%). There is little/no evidence that biostimulants decreased the vitamin C concentration in the edible portions of different crop types, in any crop type.

#### Intercropping

There is good or some evidence that intercropping increased the grain Zn concentration of maize (33%) and other crops (100%; Table 4). There was no evidence of intercropping effects on increasing grain Zn concentration in wheat (0%). Good evidence of intercropping effects on increasing grain Fe concentration was reported in wheat (100%) and maize (67%) with some evidence of 50% reported in other crops. No studies measured effects of intercropping on the concentration of Zn or Fe in the edible portions of the other specific crop types. There is little/no evidence that intercropping decreased Zn or Fe concentrations in the edible portions of wheat and other crop types, but good or some evidence in maize Zn concentration (67%) and Fe concentration (33%) was reported. There is good evidence that intercropping increased the vitamin C concentration in the edible portions of tomato (100%) and other crops (50%). There is some evidence that intercropping decreased the vitamin C concentration in the edible portions of tomato (50%) and other crops (50%). No studies measured effects of intercropping on vitamin C concentrations in the edible portions of the other specific crop types.

## Discussion

### Agronomic approaches have a role to play in crop nutrition

Findings from this scoping review showed some good evidence of increasing micronutrient concentration in crops with agronomic approaches encompassing RA. Organic inputs improved nutritional composition of crops and similarly biostimulants and intercropping, albeit based on small numbers. Examples of biostimulants with beneficial influence on crop nutrition from this study included a microbial consortium of AMF *Glomus intraradices* BEG72, *Glomus mossae* and *Trichoderma atroviride* MUCL 45632 and bacterial strains of the *Bacillus Pseudomonas* and *Arthrobacter* sps (88, 117). Evidence of increases in micronutrient concentration due to biostimulants were alluded to enhanced mycorrhizal colonization with bacterial inoculation, and improved seedling establishment (including crop rooting and vigor) which enhances micronutrient uptake (117). Intercropping increased Zn, Fe and vitamin C concentration in edible portions of cereals (maize and wheat), tomatoes and other crops. This is due to increased nutrient availability from release of compounds (including siderophores) from graminaceous cereals crops which consequently benefits the neighboring non-graminaceous crop (11, 12). Alternatively, increases in micronutrients in intercropping systems could be attributed to improved soil structure from the leguminous crop which improves retention/plant availability of micronutrients. Alternative tillage strategies employed over 2-years improved grain Zn concentration of wheat (118). However, implementation of alternative tillage strategies over a longer period did not have any effects on grain Zn concentration of wheat and maize, although significant effects were reported for heavy metals (107). Future studies could focus on effects of long-term tillage strategies (often accompanied by residue incorporation) on soil physio-chemical properties such as P accumulation, which might negatively influence uptake of essential micronutrients including Zn. Fewer studies on the effects of deficit irrigation strategies on Zn or Fe concentrations in the edible portions of most crops were reported in this study. However, there was some evidence of increases in grain Zn, and decreases in grain Fe concentration, in rice, likely reflecting complex effects of soil water content on soil micronutrient availability. Future studies could focus on impacts of irrigation strategies (i.e., alternate wetting and drying) complimented with increased organic inputs on micronutrient concentration, especially Fe which decreased in 100% of the studies.

### Strengths and weaknesses of this scoping review study

We consider that a strength of this study is the scope of the literature search, and the large numbers of papers returned during stage 1 of the search (*n* = 4,463). The choice of crop types and micronutrients included in the search were made following discussions between co-authors, representing academic and private sectors, but these terms were established *a priori* to avoid any further selection bias arising. We therefore consider this scoping study to be representative, notwithstanding the potential for selection bias in terms of the original publications.

A general weakness of this study is that we do not report the magnitude of the effects of different RA practices on crop nutritional quality for each study, nor have we used formal meta-analysis techniques. There are two main reasons: (1) for each combination of crop and RA category, the number of studies is generally small, with many studies being underpowered to detect relatively small effect sizes, and (2) where the number of studies in a category is larger (e.g., tomato under deficit irrigation; wheat with organic inputs), the diverse conditions between studies, including input types, and landscape/soil factors, mean that direct comparisons of studies would have little value. These weaknesses are discussed further in the following section.

### Potential underpowering of studies in terms of detecting small effect sizes

Relatively small improvements in the micronutrient quality of staple crops can be impactful in terms of micronutrient provisioning in food systems (14). However, effect sizes and the statistical quality of each study were not assessed within this scoping review. Notably, we did not see a power analysis reported in any of the studies. However, many studies will be underpowered to detect small effect sizes due to a small number of replications. When nutritional outcome indicators are reported from human/animal studies, a power analysis/registered trial report would be an ethical requirement of any trial design.

**Figure 3:**
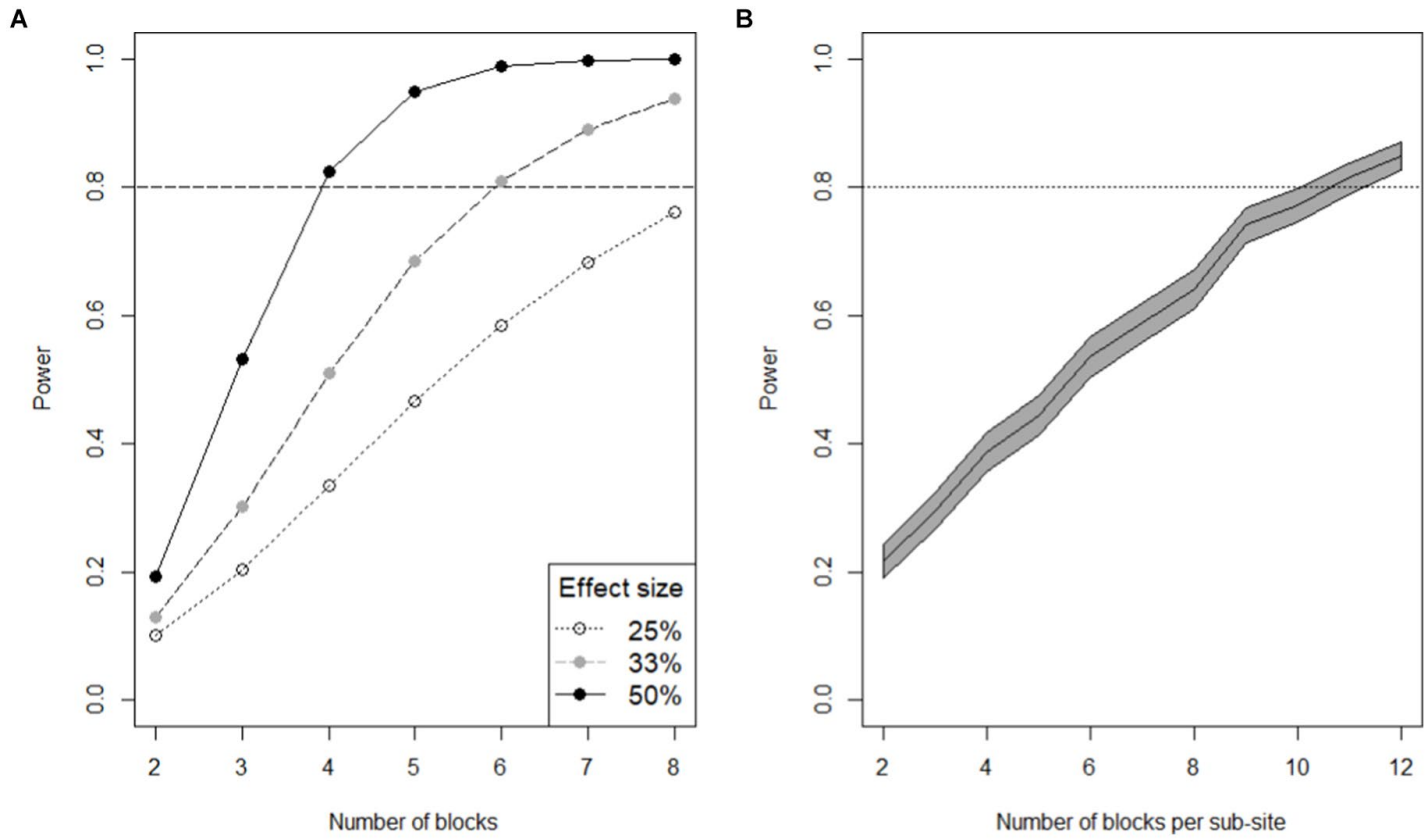
**(A)** Power analysis for a simple control/treatment experiment for an effect size of 25, 33% or 50% under soil-applied Zn fertilizers. Data are based on a treatment mean wheat grain Zn concentration of 36.9 mg kg^−1^ and a residual mean square of 35.1, as observed at a site in Pakistan; Zia et al. (120). **(B)** Power to detect a 10% effect size of soil-applied Zn fertilizer treatment on maize grain Zn concentration in Malawi; Botoman et al. (119). The grey band shows the 95% confidence interval for estimated power to detect fertilizer effects for differing numbers of blocks per sub-site (i.e., replicates). The central line is the estimated power (119).

Two studies have recently discussed the issues of small effect sizes in terms of nutritional quality responses of crops under different agronomic treatments, even when large amounts of a micronutrient are added in the form of Zn fertilizers (119, 120). For example, in Pakistan, Figure 3A illustrates that ~8 replicates would be needed to detect a 25% effect size in terms of wheat grain Zn concentration, with an ~80% experimental power, when Zn fertilizers are applied to soils. In Malawi, Figure 3B illustrates that ~10 replicate blocks, would be needed to detect a 10% difference in maize grain Zn concentration at a similar power, again, when Zn fertilizers are applied to soils. Notably, these studies were based on data from trials conducted in research station settings. For on-farm type designs, influenced by additional landscape covariates including soil type, climate, etc., appropriate replications for RA interventions may need to be larger, especially given the magnitude of an RA intervention on grain quality is often likely to be smaller than direct application of micronutrient fertilizers (14).

### The challenges of comparing crop responses across different environments and experimental conditions

Recent studies have reported large geographical differences in the micronutrient concentration (including Zn and Fe) of staple cereal grains, which is important in the context of generating robust evidence on the influence of RA approaches on crop nutritional quality. For example, in the Amhara region of Ethiopia, there was spatially correlated variation in cereal grain Zn concentration with wheat grain Zn concentration varying by more than 30% (~20–27 mg/kg) between districts (121). In Malawi, maize grain Zn concentration showed similar evidence of spatially correlated variation (27). Some of this variation was associated with soil and environmental covariates. What this means is, beyond localized sources of variation (e.g., crop variety, agronomic practices, etc.), the geographical location of a household can sometimes be the largest factor influencing the dietary intake of Zn from cereals. Combining outputs of experiments involving RA approaches, across widely different geographical locations and without considering geospatial variation, would therefore be difficult to justify.

## Conclusion

Evidence from this scoping review showed potential of RA practices to increase the concentrations of micronutrients in the edible portions of most crops. However, detecting changes in crop nutritional quality due to RA agronomic approaches is inherently challenging, due to potentially small effect sizes and the inherent variation in crop micronutrient composition due to other factors (e.g. crop yield and variety, soil type, and other landscape factors). Whilst the effect size of an RA agronomic approach is relatively small, the population-level health benefits through improved dietary micronutrient supply could potentially be large. This study was limited due to a lack of a formal meta-analyses to statistically quantify the magnitude of the effects of different RA practices on crop nutritional quality. This is largely due to small sample sizes within each RA and crop (+ nutrient) category and diverse conditions (i.e. input types and environmental factors), impeding a more direct comparison of studies. Future research should include appropriate experimental designs to test RA-informed interventions from which potential crop nutritional co-benefits (or trade-offs) might arise. Improvements in on-farm surveillance and nutritional diagnostics and a greater appreciation of the value of including crop nutritional quality metrics, potentially plays a key role in understanding linkages between RA, human nutrition, and human health. Additionally, linkages to potential nutritional outcomes within agronomic studies could be established, particularly when combined with other food system interventions, including biofortification of crops through breeding and/or the use of micronutrient-based fertilizers.

## Author contributions

MGM-K, RML, SR, AE, and MRB contributed to the conceptualization of the paper. MGM-K, RML, and MRB conducted the initial screening of abstracts. MGM-K and MRB conducted the second screening of abstracts and wrote the initial draft of the manuscript. MGM-K, EJMJ, and MRB conducted the full review of 367 records included in the review. All authors contributed to the article and approved the submitted version.

## Funding

Funding was received from Unilever United Kingdom Central Resources Limited to conduct a “Review of agricultural practices that lead to improved nutrient content of crops (Regenerative Nutrition).”

## Conflict of interest

SR and AE are employees of Unilever.

The authors declare that this study received funding from Unilever UK Central Resources Limited. The Funder had the following involvement in the study: study design and boundaries (micronutrients and crops to include, agricultural practices to include/exclude), writing of this article and the decision to publish. The funder was not directly involved in collection, analysis and interpretation of data.

## Publisher’s note

All claims expressed in this article are solely those of the authors and do not necessarily represent those of their affiliated organizations, or those of the publisher, the editors and the reviewers. Any product that may be evaluated in this article, or claim that may be made by its manufacturer, is not guaranteed or endorsed by the publisher.

## Footnotes

^1^Not limited to certified organic agriculture practices.

^2^https://www.zotero.org/groups/4531670/unilever_scoping_analysis/library
