# Supplementary material for: Do agronomic approaches aligned to regenerative agriculture improve the micronutrient concentrations of edible portions of crops? A scoping review of evidence
Source: Front Nutr. 2023 Jul 12;10:1078667. doi: 10.3389/fnut.2023.1078667 (PMC10371419; doi:10.3389/fnut.2023.1078667)
Supplement: Supplementary file 1 [file Data_Sheet_1.docx]

**Supplementary Tables**

**Table 1. Studies reporting effects of Regenerative Agriculture on various nutrient concentrations in tomato**

| **Primary regenerative ag. strategy reported** | **Micronutrient [evidence of direction of change, ↔︎,↑,↓,↕︎]** | **Reference*** |
| --- | --- | --- |
|  |  |  |
| Organic Inputs | vit-C [↑] | Duan et al., [6] |
|  | vit-C [↑], lycopene [↑] | Wang et al., [7] |
|  | vit-C [↓], lycopene [↔︎], selenium [↑] | Huang et al., [8] |
|  | vit-C [↔︎] | She et al., [10] |
|  | vit-C [↑] | Guo et al., [12] |
|  | Calcium [↔︎] | Colla et al., [15] |
|  | vit-C [↔︎], lycopene [↔︎] | Wu et al., [18] |
|  | vit-C [↑] | Zhang et al., [21] |
|  | vit-C [↑], beta-carotene [↑] | Yu et al., [22] |
|  | vit-C [↔︎] | Majkowska-Gadomska et al., [24] |
|  | calcium [↑], copper [↑], iron [↑], zinc [↑] | Khan et al., [32] |
|  | vit-C [↔︎], lycopene [↑], carotenoids [↑] | Turhan and Ozmen [36] |
|  | vit-C [↔︎] | Qahraman et al., [40] |
|  | vit-C [↔︎] | Ece and Uysal [41] |
|  | vit-C [↑] | Jin et al., [42] |
|  | vit-C [↑] | Özer [47] |
|  | vit-C [↔︎] | Rady [52] |
|  | vit-C [↑] | Abduli et al., [53] |
|  | calcium [↑], magnesium [↔︎] | Tonfack et al., [61] |
|  | vit-C [↔︎], lycopene [↑] | Mukherjee et al., [62] |
|  | vit-C [↑] | Song et al., [64] |
|  | calcium [↓], magnesium [↑] | Galieni et al., [65] |
|  | vit-C [↔︎] | Tüzel et al., [67] |
|  | vit-C [↔︎], carotenoids [↓] | Ceglie et al., [69] |
|  | calcium [↔︎], copper [↔︎], iron [↔︎], magnesium [↔︎], manganese [↔︎], zinc [↔︎] | Warman [79] |
|  | vit-C [↑], carotenoids [↑] | Dinu et al., [84] |
|  | vit-C [↓], lycopene [↓], beta-carotene [↓], tocopherols [↑] | Petropoulos et al., [92] |
|  | vit-C [↑], polyphenols [↑] | Nabaei et al., [94] |
|  | vit-C [↔︎], lycopene [↔︎], beta-carotene [↔︎], carotenoids [↔︎] | Rosa-Martinez et al., [99] |
|  | vit-C [↔︎], calcium [↔︎], copper [↑], iron [↑], magnesium [↔︎], manganese [↔︎], zinc [↑] | Polat et al., [104] |
|  |  |  |
| Irrigation | vit-C [↑] | Guida et al., [4] |
|  | calcium [↔︎] | Taylor et al., [13] |
|  | vit-C [↑], lycopene [↑], phenolics [↑] | Helyes et al., [23] |
|  | vit-C [↑] | Al-Selwey et al., [26] |
|  | vit-C [↔︎], lycopene [↔︎] | Helyes et al., [30] |
|  | lycopene [↑],beta-carotene [↔︎], phenolics [↑] | Pék et al., [31] |
|  | vit-C [↓], lycopene [↑], carotenoids [↑] | Turhan et al., [48] |
|  | vit-C [↑] | Shao et al., [58] |
|  | vit-C [↑] | Nangare et al., [59] |
|  | vit-C [↑], lycopene [↔︎], beta-carotene [↔︎] | Martí et al., [63] |
|  | vit-C [↑] | Chen et al., [72] |
|  | vit-C [↑] | Abdel-Razzak et al., [74] |
|  | vit-C [↑], lycopene [↑] | Du et al., [75] |
|  | lycopene [↓] | Liu et al., [76] |
|  | vit-C [↔︎] | Al-Harbi et al., [77] |
|  | vit-C [↑], lycopene [↔︎] | Wu et al., [78] |
|  | vit-C [↑], lycopene [↕︎] | Wang et al., [81] |
|  | carotenoids [↔︎] | Pokluda et al., [86] |
|  | lycopene [↑] | Helyes et al., [89] |
|  | lycopene [↑], beta-carotene [↔︎], flavonoids [↑], tocopherols [↕︎], phenolics [↑] | Helyes et al., [93] |
|  | vit-C [↓], lycopene [↔︎], phenolics [↑] | Helyes et al., [96] |
|  | vit-C [↑], lycopene [↑] | Samui et al., [106] |
|  | vit-C [↑] | Cui et al., [107] |
|  |  |  |
| Biostimulants (PGPBs) | vit-C [↑] | Tiyagi et al., [56] |
|  | vit-C [↑], lycopene [↕︎], beta-carotene [↕︎], carotenoids [↕︎] | Le et al., [87] |
|  |  |  |
| Intercropping | vit-C [↕︎] | Demir and Polat [49] (with lettuce) |
|  | vit-C [↑] | Liu et al., [60] (with garlic) |

*Numbers in the author column refer to alphabetical order of paper title in the Zotero subfolders.

**Table 2. Studies reporting effects of Regenerative Agriculture on secondary and micronutrient concentrations in wheat**

| **Primary regenerative ag. strategy reported** | **Micronutrient [evidence of direction of change, ↔︎, ↑, ↓, ↕︎]** | ***Reference** |
| --- | --- | --- |
| Organic Inputs | Fe [↑︎], Zn [↑] | Barlog et al., [14] |
|  | Mg [↑], Ca [↔︎] | Bowszys et al., [60] |
|  | Fe [↔︎], Ca [↔︎], Mg [↔︎] | Chauhan et al., [23] |
|  | Zn [↑] | Chen et al., [79] |
|  | Zn [↓] | Cooper et al., [28] |
|  | Se [↓] | Dhillon et al., [72] |
|  | Fe [↔︎], Zn [↑], Mg [↔︎] | Fan et al., [41] |
|  | Se [↔︎] | Fan et al., [48] |
|  | Zn [↕︎], Mg [↓] | Gondek [20] |
|  | Zn [↑] | Gruter et al., [46] |
|  | Zn [↔︎] | Hamner and Kirchmann [88] |
|  | Fe [↑], Zn [↑] | Khaliq et al., [75] |
|  | Vit A [↑], Vit E [↕︎] | Konopka et al., [13] |
|  | Fe [↔︎], Zn [↔︎] | Kumar et al., [5] |
|  | Fe [↔︎], Zn [↑] | Puniya et al., [24] |
|  | Fe [↔︎], Zn [↔︎] | Randhawa et al., [49] |
|  | Fe [↔︎], Zn [↑], Ca [↔︎], Mg [↔︎] | Ryan et al., [44] |
|  | Zn [↑] | Saha et al., [11] |
|  | Se [↓] | Sharma et al., [27] NB seleniferous soils |
|  | Fe [↔︎], Zn [↔︎] | Shivay et al., [77] |
|  | Fe [↔︎], Zn [↔︎] | Shivay et al., [77] |
|  | Zn [↑] | Soltani et al., [81] |
|  | Fe [↔︎], Zn [↔︎] | Stepien and Wojtkowiak [25] |
|  | Fe [↓], Zn [↔︎] | Stepien and Wojtkowiak [25] |
|  | Fe [↑], Zn [↑], Ca [↔︎] | Thomas et al., [80] |
|  | Ca [↓], Mg [↑] | Tlustos et al., [64] |
|  | Fe [↔︎], Zn [↔︎] | Turk et al., [65] |
|  | Fe [↔︎], Zn [↑] | Turmel et al., [82] |
|  | Fe [↓], Zn [↔︎], Ca [↔︎] | Wang et al., [22] |
|  | Se [↓] | Wang et al., [51] |
|  | Mg [↓] | Wilczewski et al., [26] |
|  | Fe [↑], Zn [↓], Ca [↑], Mg [↔︎] | Woźniak [94] |
|  | Vit C [↑] | Woźniak et al., [76] |
|  | Zn [↓] | Wu et al., [7] |
|  | Fe [↔︎], Zn [↔︎] | Zhang et al., [58] |
|  |  |  |
| Intercropping | Fe [↑], Zn [↔︎] | Gunes et al., [62] |
|  |  |  |
| Tillage | Fe [↔︎], Zn [↔︎], Ca [↔︎] | Galieni et al., [63] |
|  | Mg [↔︎] | Jaskiewicz [10] |
|  | Fe [↔︎], Zn [↔︎], Mg [↓] | Kraska [15] |
|  | Fe [↔︎] | Shahane et al., [39] |
|  | Fe [↔︎], Zn [↕], Ca [↔︎], Mg [↓] | Woźniak [94] |
|  |  |  |
| Biostimulants (AMF) | Fe [↑], Zn [↑] | Colla et al., [12] |
| AMF | Zn [↑] | Farahbakhsh and Khasse Sirjani [40] |
| Bacterial strains (*Bacillus* *Pseudomonas* and *Arthrobacter* sps) | Fe [↑], Zn [↔︎], Ca [↕], Mg [↔︎] | Ogut and Er [61] |
| Bacterial strain *(Azotobacter* sp*)* | Fe [↔︎], Zn [↔︎] | Shivay et al., [77] |
| Amino acids | Fe [↓], Zn [↑], Ca [↓], Mg [↓] | Souza et al., [95] |
| PGPB and AMF | Fe [↑], Zn [↑] | Yadav et al., [2] |
|  |  |  |

*Numbers in the author column refer to alphabetical order of paper title in the Zotero subfolders.

**Table 3. Studies reporting effects of Regenerative Agriculture on secondary and micronutrient concentrations in rice**

| **Primary regenerative ag. strategy reported** | **Micronutrient [evidence of direction of change, ↔︎,↑,↓, ↕︎]** | **Reference*** |
| --- | --- | --- |
| Organic fertilisers or amendments | Zn [↑] | Hussain et al., [1] |
|  | Zn [↑], Fe [↓] | Li et al., [4] |
|  | Zn [↑] | Wan et al., [10] |
|  | Zn [↑], Fe [↑] | Bhargavi et al., [11] |
|  | Zn [↑] | Singh and Shivay [12] |
|  | Zn [↑], Fe [↑] | Saha et al., [15] |
|  | Zn [↑], Fe [↑] | Patra et al., [19] |
|  | Zn [↕︎], Fe [↕︎] | Sharma et al., [22] |
|  | Zn [↑], Fe [↑] | Ramzani et al., [23] |
|  | Zn [↑], Fe [↕︎], Mg [↕︎], Ca [↕︎] | Liu et al., [25] |
|  | Zn [↑], Fe [↓] | Velmurugan and Swarnam [28] |
|  | Zn [↑], Fe [↑] | Saha et al., [29] |
|  | Zn [↑] | Mishra et al., [30] |
|  | Zn [↑] | Zhang et al., [35] |
|  | Zn [↑], Fe [↕︎] | Sha et al., [37] |
|  | Zn [↔︎] | Pooniya et al., [40] |
|  |  |  |
| Irrigation | Zn [↔︎], Fe [↓] | Norton et al., [3] |
|  | Fe [↓] | Talkudar et al., [17] |
|  | Zn [↑] | Lan et al., [18] |
|  | Zn [↓] | Rehman et al., [24] |
|  | Zn [↑], Se [↑] | Martinez-Eixarch et al., [26] |
|  | Fe [↓], Se [↓], Zn [↓] | Xu et al., [33] |
|  | Zn [↑] | Xu et al., [39] |

*Numbers in the author column refer to alphabetical order of paper title in the Zotero subfolders.

**Table 4. Studies reporting effects of Regenerative Agriculture on secondary and micronutrient concentrations in maize.**

| **Primary regenerative ag. strategy reported** | **Notes** | **Micronutrient [evidence of direction of change, ↔︎,↑,↓, ↕︎]** | **Reference*** |
| --- | --- | --- | --- |
| Organic Inputs | Baby corn | Fe [↑], Zn [↑], vit-C [↑] | Babu et al., [9] |
|  | Maize | Zn [↑], Fe [↑] | Mubarak et al., [25] |
|  | Maize (following wheat) | Ca [↑], Mg [↑], Zn [↑], Fe [↑] | Thakur et al., [12] |
|  | Maize-Wheat cropping | Zn [↑], Fe [↑] | Kumar et al., [19] |
| Intercropping | Fodder maize (with Common beans and spring wheat) | Zn [↓] | Glowacka [10] |
|  | Dent maize (with blue lupin and oat) | Fe [↑] | Glowacka [22] |
|  | Dent maize (with blue lupin and oat) | Zn [↑], Fe [↑] | Glowacka [23] |
|  | Dent maize (with blue lupin and oat) | Mg [↓], Ca [↑] | Glowacka [3] |
|  | Maize (with Faba bean, Chickpea, Soybean and Turnip) | Zn [**↓**], Fe [**↓**] | Xia et al., [11] |
| Tillage | Maize (following Wheat-soyabean continuous sequence) | Zn [↔︎] | Lavado et al., [14] |

*Numbers in the author column refer to alphabetical order of paper title in the Zotero subfolders.

**Table 5. Studies reporting effects of Regenerative Agriculture on secondary and micronutrient concentrations in pulses**

| **Primary regenerative ag. strategy reported** | **Crop type** | **Micronutrient [evidence of direction of change, ↔︎,↑,↓, ↕︎]** | **Reference*** |
| --- | --- | --- | --- |
|  |  |  |  |
| Organic Inputs | Snap bead (Phaseolus vulgaris) | Zn [↔︎] | Wen et al., [1] |
|  | Faba bean (Vicia faba) | Cu [↑], Fe [↑], Mn [↑], Zn [↑] | El-Husseiny et al., [10] |
|  | Bean (Phaseolus vulgaris, 2 varieties) | Ca [↔︎], Cu [↔︎], Fe [↔︎], Mg [↔︎], Mn [↔︎], Zn [↔︎] | Warman [16] |
|  | Pea (Pisum sativum) | Ca [↔︎], Cu [↔︎], Fe [↔︎], Mg [↔︎], Mn [↔︎], Zn [↔︎], vit-C [↔︎] | Fjelkner-Modig [19] |
|  | Cowpea (Vigna unguiculata) | Zn [↑] | Manzeke et al., [22] |
|  |  |  |  |
| Intercropping | Pea (Pisum sativum) [with oat; Avena sativa] | Ca [↔︎], Mg [↔︎] | Neugschwandtner and Kaul [4] |
|  |  |  |  |
| Biostimulants (AMF) | Pea (Pisum sativum) | vit-C [↑] | Yadav et al., [8] |
|  |  |  |  |

*Numbers in the author column refer to alphabetical order of paper title in the Zotero subfolders.

**Table 6. Studies reporting effects of Regenerative Agriculture on various nutrient concentrations in alliums**

| **Primary regenerative ag. strategy reported** | **Crop type** | **Micronutrient [evidence of direction of change, ↔︎,↑,↓, ↕︎]** | **Reference*** |
| --- | --- | --- | --- |
|  |  |  |  |
| Organic Inputs | Leek | vit-C [↑] | Lundegårdh et al., [9] |
|  | Onion | vit-C [↑], Ca [↑], Mg [↑], Fe [↑], Zn [↑] | Ncayiyana et al., [1] |
|  | Onion | vit-C [↔︎], Cu [↓], Fe [↓], Mn [↓], Zn [↔︎], flavonoids [↑], phenolics [↑] | Thangasamy et al., [2] |
|  | Onion | vit-C [↑] | Latha and Sharanappa [3] |
|  | Onion | Ca [↔︎], Cu [↔︎], Fe [↔︎], Mg [↔︎], Mn [↔︎], Zn [↔︎] | Yoldas et al., [4] |
|  | Onion | Ca [↔︎], Mg [↔︎], flavonoids [↔︎], phenolics [↔︎] | Lee et al., [6] |
|  | Onion | Ca [↔︎], Mg [↔︎] | Lee et al., [8] |
|  | Onion | Ca [↔︎], Cu [↔︎], Fe [↔︎], Mg [↔︎], Mn [↔︎], Zn [↔︎] | Warman [15] |
|  | Onion | Se [↓] | Stavridou et al., [16] |
|  | Onion | vit-C [↔︎], Ca [↔︎], Cu [↔︎], Fe [↔︎], Mg [↔︎], Mn [↔︎], Zn [↔︎] | Fjelkner-Modig et al., [20] |
|  |  |  |  |
| Biostimulants (AMF) | Garlic | vit-C [↔︎], Ca [↑], Cu [↔︎], Fe [↔︎], Mg [↑], Mn [↔︎], Se [↑], Zn [↔︎], flavonoids [↔︎], phenolics [↔︎] | Golubkina et al., [7a] |
|  | Onion | vit-C [↔︎], Ca [↑], Cu [↑], Fe [↑], Mg [↔︎], Mn [↔︎], Se [↑], Zn [↔︎], flavonoids [↔︎], phenolics [↑] | Golubkina et al., [7b] |
|  | Shallot | vit-C [↑], Ca [↑], Cu [↑], Fe [↑], Mg [↑], Mn [↑], Se [↑], Zn [↑], flavonoids [↔︎], phenolics [↔︎] | Golubkina et al., [5] |

*Numbers in the author column refer to alphabetical order of paper title in the Zotero subfolders.

**Table 7. Studies reporting effects of Regenerative Agriculture on various nutrient concentrations in other crops**

| **Primary regenerative ag. strategy reported** | **Crop type** | **Micronutrient [evidence of direction of change, ↔︎,↑,↓, ↕︎]** | **Reference*** |
| --- | --- | --- | --- |
|  |  |  |  |
| Organic Inputs | Barley | Ca [↔︎], Cu [↔︎], Fe [↓], Mg [↔︎], Mn [↔︎], Zn [↔︎] | Hejcman et al., [17] |
|  | Cabbage | Se [↔︎] | Stavridou et al., [48] |
|  | Cabbage | vit-C [↔︎], Ca [↔︎], Cu [↔︎], Fe [↔︎], Mg [↔︎], Mn [↔︎], Zn [↔︎] | Fjelkner-Modig et al., [51a] |
|  | Carrot | Ca [↓] | Veladžić et al., [3] |
|  | Carrot | Ca [↔︎], Cu [↔︎], Fe [↔︎] | Zdravković et al., [20] |
|  | Carrot | vit-C [↔︎], beta-carotene-C [↔︎] | Mukherjee et al., [27a] |
|  | Carrot | vit-C [↔︎], beta-carotene-C [↔︎], Ca [↔︎], Mg [↔︎], | Kaack et al., [39] |
|  | Carrot | Ca [↔︎], Cu [↔︎], Fe [↔︎], Mg [↔︎], Mn [↔︎], Zn [↔︎] | Warman [47b] |
|  | Carrot | vit-C [↔︎], Ca [↔︎], Cu [↔︎], Fe [↔︎], Mg [↔︎], Mn [↔︎], Zn [↔︎] | Fjelkner-Modig et al., [51b] |
|  | Jute mallow | Cu [↑], Fe [↔︎], Mn [↔︎], Zn [↔︎] | Oguntade et al., [5] |
|  | Korean ginseng | Vit-E [↑] | Chung et al., [22] |
|  | Lettuce | Zn [↔︎] | Wen et al., [1] |
|  | Pearl millet | Fe [↑], Zn [↑] | Bana et al., [31a] |
|  | Pepper | Ca [↔︎], Cu [↑], Fe [↔︎], Mg [↔︎], Mn [↔︎], Zn [↑] | Hernández-Aranda et al., [10] |
|  | Pepper | vit-C [↔︎] | Mukherjee et al., [27b] |
|  | Pepper | Ca [↔︎], Cu [↔︎], Fe [↔︎], Mg [↔︎], Mn [↔︎], Zn [↔︎] | Warman [47d] |
|  | Potato | Ca [↔︎], Cu [↔︎], Mg [↔︎], Mn [↔︎], Zn [↔︎] | Šrek et al., [16] |
|  | Potato | Ca [↔︎], Mg [↔︎] | Tein et al., [25] |
|  | Potato | vit-C [↔︎], Ca [↔︎], Cu [↔︎], Fe [↔︎], Mg [↔︎], Mn [↔︎], Zn [↔︎] | Fjelkner-Modig et al., [51c] |
|  | Strawberry | vit-C [↑] | Balci et al., [24] |
|  | Cocoyam | Ca [↔︎], Mg [↔︎] | Iwuagwu et al., [46] |
|  |  |  |  |
| Intercropping | Carrot | vit-C [↔︎] | de Araujo Ferreira et al., [43] |
|  | Carrot | Ca [↑], Cu [↑], Mg [↑] | Majkowska-Gadomska et al., [53] |
|  | Lettuce | vit-C [↕︎] | Demir and Polat [23] |
|  | Oat | Ca [↓], Mg [↓] | Neugschwandtner and Kaul [13] |
|  | Cabbage | Ca [↔︎], Mg [↔︎], Fe [↔︎] | Guvenc and Yildirim [28] |
|  | Pearl millet | Fe [↑], Zn [↑] | Bana [31b] |
|  |  |  |  |
| Biostimulants (plant growth promoting bacteria) | Potato | vit-C [↑] | Volkogon et al., [8] |
|  |  |  |  |
| Irrigation | Pepper | Carotenoids [↔︎] | Pokluda et al., [50a] |
|  | Lettuce | Carotenoids [↔︎] | Pokluda et al., [50b] |

*Numbers in the author column refer to alphabetical order of paper title in the Zotero subfolders.
